# Tolerogenic Role of Myeloid Suppressor Cells in Organ Transplantation

**DOI:** 10.3389/fimmu.2019.00374

**Published:** 2019-03-06

**Authors:** Jordi Ochando, Patricia Conde, Alberto Utrero-Rico, Estela Paz-Artal

**Affiliations:** ^1^Department of Medicine, Icahn School of Medicine at Mount Sinai, New York, NY, United States; ^2^Immunología de Trasplantes, Centro Nacional de Microbiología, Instituto de Salud Carlos III, Madrid, Spain; ^3^Grupo de Inmunodeficiencias e Inmunología del Trasplante, Instituto de Investigación Sanitaria Hospital 12 de Octubre (imas12), Madrid, Spain; ^4^School of Medicine, Complutense University, Madrid, Spain

**Keywords:** MDSC, immune tolerance, transplantation, immunophenotyping, myeloid cells

## Abstract

Myeloid-derived suppressor cells (MDSC) are a heterogeneous population of immature cells of myeloid origin with a specific immune inhibitory function that negatively regulates the adaptive immune response. Since MDSC participate in the promotion of tolerance in the context of organ transplantation, therapeutic strategies that regulate the induction and development of MDSC have been the center of scientist attention. Here we review literature regarding induction of MDSC with demonstrated suppressive function among different types of allografts and their mechanism of action. While manipulation of MDSC represents a potential therapeutic approach for the promotion of donor specific tolerance in solid organ transplantation, further characterization of their specific phenotype, which distinguishes MDSC from non-suppressive myeloid cells, and detailed evaluation of the inhibitory mechanism that determines their suppressive function, is necessary for the realistic application of MDSC as biomarkers in health and disease and their potential use as immune cell therapy in organ transplantation.

## Introduction

Myelopoiesis is a regular process where the cells of the mononuclear phagocyte system (MPS) originate from common myeloid precursors (CMP) leading to monocytes, macrophages and dendritic cells (DC) under steady state. Under acute pathological conditions, myeloid cells respond to immunogenic signals like PAMPs (pathogen-associated molecular patterns) or DAMPs (damage-associated molecular patterns) showing multiple protective immune functions such as phagocytosis, pro-inflammatory cytokine secretion and activation of T cells. Under chronic pathological conditions, such as persistent inflammation or certain malignancies, myeloid cells are stimulated by continue immunogenic signals that have important effects on cell differentiation (1). The standard pathway for CMP cells differentiation is inhibited and the myelopoiesis is altered under chronic inflammation, which results in undifferentiated myeloid cells (2). Myeloid derived suppressor cells (MDSC) represent a mixture of myeloid progenitor cells at different stages of differentiation that may develop into macrophages, DC or granulocytes depending the microenvironment (3, 4).

Since the discovery of MDSC there has been an effort to define their heterogeneity, origin and function beyond cancer relates studies. The scientific interest of these cells have been spread through many fields where the immune system is altered due to chronic pathological conditions, such as graft vs. host disease (GvHD), organ transplantation, infection, and autoimmune diseases. To better define the heterogeneity, murine MDSC were initially defined as myeloid cells expressing CD11b and GR-1. Based on these two markers and their morphology, MDSC were divided into two major groups: Granulocytic MDSC (G-MDSC) and monocytic MDSC (M-MDSC). Several studies included the differential expression of inducible nitric oxide synthase (iNOS) and arginase (Arg) and subdivided MDSC population based on the expression of Ly6C and Ly6G (5). CD11b+Ly6ChiLy6G- MDSC have a monocytic-like morphology express nitric oxide synthase 2 (NOS2), have increased T cell suppressive activity and are identified as M-MDSC. In contrast, CD11b+Ly6ClowLy6G+ MDSC have a granulocyte-like morphology and express high levels of arginase type 1 (Arg1) (5). Some authors recommend the term polymorphonuclear (PMN)-MDSC instead of G-MDSC attending to the differences in the phenotype from steady-state neutrophils. PMN-MDSC show less granules, reduced CD16 and CD62L and increased Arg1, peroxynitrite and CD11b expression (6). Additional MDSC populations have been described based on the intensity of the Gr-1 gene expression as Gr-1lo, Gr-1int, and Gr-1hi (7). Based on recent findings showing differences in modulation of the cell death pathway, the anti-apoptotic markers c-FLIP and MCL-1 could be also of help to respectively distinguish M-MDSC and granulocytic MDSC subsets. Different from granulocytic MDSC, the continuous expression of c-FLIP is needed by M-MDSC survival and function and defines them as the dominant immunosuppressive subset. This observation points out modulation of c-FLIP in monocytes to promote or block immunosuppressive cells for therapy purposes (8, 9).

The overlapping expression of phenotypic markers makes also difficult to distinguish MDSC from tumor-associated macrophages (TAM) and tumor-associated neutrophils (TAN). Mouse M-MDSC are phenotypically described as CD11b+Ly6ChiLy6G- myeloid cells expressing low levels of F4/80, while TAM express high levels of F4/80 (4). Human MDSC were initially described in cancer patients as lineage negative CD34+ cells (10). Thereafter, other myeloid markers such as the human leucocyte antigen (HLA)-DR was identified in a renal cell carcinoma study to define human MDSC as CD33+CD11b+HLA-DR- (11). Additionally, the use of CD14 expression is accepted for human M-MDSC, although it is still controversial for PMN-MDSC since granulocytes express low levels of this marker. As a result, M-MDSC are defined as CD33+CD11b+HLA-DR-CD14+ while PMN-MDSC are defined as CD33+CD11b+CD15+CD66b+ (6). Recently, another MDSC subpopulation was described as CD33+CD11b+HLA-DR-CD14-CD15-CD66b-, including a mixture of immature cells named early stage MDSC (e-MDSC). Recent findings suggest that PMN- and M-MDSC are the most potent immunosuppressive cells while e-MDSC show less Arg1 and iNOS amounts and may not inhibit T cells proliferation. More studies are needed to understand if e-MDSC are true MDSC precursors and evaluate their clinical significance (12). It is also recommended the use of additional phenotypic markers, such as CD62L, CD16 and the vascular endothelial growth-factor receptor1 (VEGFR1) to better define human MDSC (13).

Phenotypic characterization of MDSC remains controversial as MDSC are described as myeloid cells in different stages of differentiation associated with immune-regulatory molecules and receptors (Arg1, NOS2/NO, NOX2/ROS, PD-L1, and VEGF2), transcription factors (S100a8 and STAT3) and cytokines (IL-10, TGFβ and IL4-R) (6). Since phenotypic characterization of MDSC is still debatable, MDSC are better defined as potent immunosuppressive myeloid cells characterized by less phagocytic activity or the production of high levels of reactive oxygen and nitrogen species and anti-inflammatory cytokines (14). The capacity to modulate T cells activity is the most often used immune suppressive feature of MDSC, which is also associated with their increased capacity to induce T cell apoptosis (15) and expansion of regulatory T cells (Treg) (16). Although immune modulation of T conventional cell activity is probably the main reported function of MDSC, the interaction between MDSC and other immune cells has been described in recent years. These include suppression of the B cells (17), dysregulation of T follicular helper cells (18, 19), loss of natural killer cell (NK) function (11) and suppression of DC development (20).

Focusing on the mechanism of MDSC induction, inhibitors of the mammalian target of rapamycin (mTOR), which represents a major immunosuppressive drugs for organ transplant recipients (21), plays a crucial role in promoting the development of MDSC. Using an immunological hepatic injury model, it was demonstrated rapamycin served as a functional immune modulator of CD11b^+^Gr1^+^ MDSC (22). Mechanistically, the authors demonstrated that mTOR down-regulation promotes CD11b^+^Gr1^+^Ly6C^hi^iNOS^+^ M-MDSC recruitment to the inflammatory site that produced NO for tissue repair. Rapamycin also enhanced the suppressive function in murine PMN-MDSC after bone marrow transplantation, via up-regulation of Arg1 and iNOS (23). However, the effects of rapamycin and mTOR inhibition on MDSC remains controversial, as transgenic mice with a myeloid-specific deletion of mTOR display a decreased the number of M-MDSC *in vivo* after skin allograft transplantation (24).

## MDSC in Organ Transplantation

The mononuclear phagocyte system (MPS), comprising DC, monocytes and macrophages, is implicated in many immunological mechanisms occurring during recognition of the non-self and the alloimmune response against the transplanted organ (25). Recipient DC infiltrate the allograft and form cognate contacts with T cells promoting effector T cell mediated rejection (26). In addition, donor DC derived exosomes promote an alloimmune response against the allograft by transferring functional MHC molecules to recipient DC (27). Acquisition of exosomes activates recipient DC that present donor MHC molecules to alloreactive T cells promoting T cell immunity. Monocytes also play a critical role in organ transplantation as they mediate the immune response against allogeneic non-self (28) and initiate allograft rejection by inducing T cell mediated immune responses (29). Macrophages act as effectors of tissue damage in acute renal allograft rejection (30) and represent the majority of cells that infiltrate an allograft under severe rejecting conditions (31). Mechanistically, activated graft infiltrating macrophages increase their aerobic glycolysis metabolism and secrete pro-inflammatory cytokines associated with acute rejection (32). In addition to the MPS, neutrophils also play a critical role during organ rejection. The Lakkis laboratory demonstrated that depletion of neutrophils with anti-Ly6G significantly decreased inflammatory alloresponses (28). This is consistent with previous observations, which suggested that early neutrophil influx into the transplanted allograft favors organ rejection (33). Mechanistically, neutrophils may contribute to allograft rejection by different pathways that include the secretion of inflammatory cytokines (34), B cell stimulation (35) and through antigen presentation to T cells (36).

**Figure 1:**
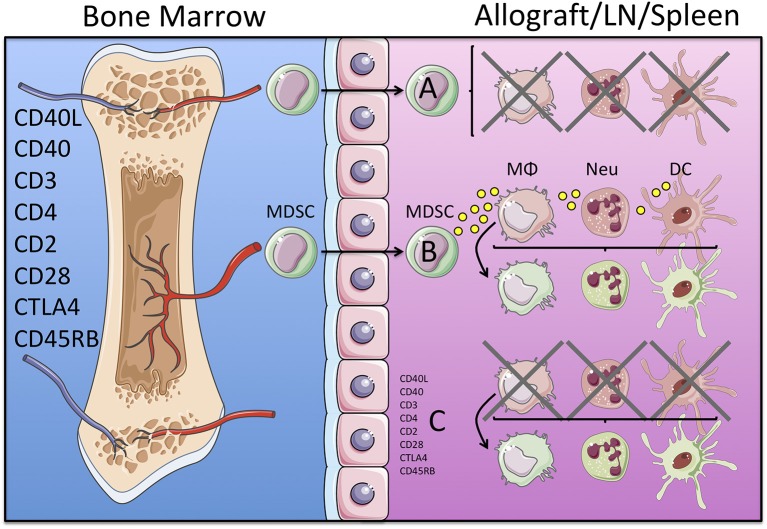
Potential mechanisms of immune regulation mediated by MDSC in organ transplantation. Induction of transplantation tolerance in experimental murine models is achieved by targeting TCR and co-stimulatory blockade with monoclonal antibodies. These therapeutic treatments may induce the development of an MDSC precursor that leaves the bone marrow and may migrate into the allograft, lymph node (LN) and/or the spleen. Once in the tissue MDSC may mediate direct inhibition of immunogenic myeloid cells (macrophages, neutrophils and dendritic cells in red), as depicted in **(A)**; or secrete cytokines and growth factors that convert immunogenic (red) into tolerogenic (green) myeloid cells, as depicted in **(B)**. Alternatively, both processes (direct inhibition of immunogenic and/or conversion into tolerogenic myeloid cells) may be a direct effect of the tolerogenic regimen (monoclonal antibodies) independently of the MSDC, as depicted in **(C)**.

Since DC, monocytes, macrophages and neutrophils all the myeloid contribute to organ transplant rejection, MDSC must therefore prevent their immunogenicity against the allograft. Consequently, therapeutic protocols that prolong organ transplant survival may induce the development of MDSC, which inhibit myeloid cell derived graft reactive immune responses, such as antigen presentation and lymphocyte activation. Alternatively, experimental approaches that promote organ transplant acceptance may skew the differentiation of immunogenic DC, monocyte, macrophage and neutrophil precursors toward M-MDSC and G-MDSC favoring immune tolerance (Figure 1). Below we describe the role of MDSC in different organ transplant settings.

### Kidney Transplantation

Vanhove's laboratory was the first to report the role of MDSC in kidney transplant recipient rats (37). In this experimental model, tolerance was induced by a costimulatory blockade with anti CD28 antibody. Myeloid cells expressing CD11b^+^CD80/86^+^Sirpα^+^ accumulated in the recipient allograft and were defined as MDSC for the first time in the context of organ transplantation. This study indicated that CD11b^+^Sirpα^+^ MDSC isolated from blood and bone marrow were able to suppress proliferation of anti CD3 anti-CD28 stimulated T cells. This suppressive mechanism of tolerance was in part mediated by iNOS, which was upregulated in graft infiltrating MDSC and by blood MDSC upon co-culture with activated effector T cells but not in Treg. The mechanistic role of NO in MDSC-mediated suppression was initially described by Mazzoni and colleagues using a NO synthase knockout mice (38). The authors demonstrated that CD11b^+^Gr-1^+^ MDSC from the spleens of immunosuppressed mice inhibit T cell proliferation in a NO-dependent manner, in response to signals from activated T cells that included IFN-γ. Another report from Vanhove's laboratory indicated that secretion of CCL5 by graft infiltrating MDSC was responsible for the accumulation of Treg into tolerized kidney allografts (39). In subsequent studies, Dilek and colleagues analyzed blood MDSC gene expression from kidney recipient showing that CCL5 was strongly downregulated after tolerant regimen. The amount of intra graft CCL5 protein was unchanged (40). The results indicate that a gradient of CCL5 between the graft and peripheral blood might contribute to the intra graft localization of Treg in tolerant recipients controlled by MDSC.

In the clinical setting, Hock and coworkers showed significantly increased frequencies of total MDSC (CD33^pos^ HLA-DR^neg^ cells into the blood low density fraction), as well as both CD14^pos^ and CD14^neg^-MDSC subsets in renal transplant recipients relative to normal donors. MDSC subsets frequencies and MDSC/DC ratios were higher in kidney recipients with or without current or prior squamous cell carcinoma than in healthy controls. *In vitro*, fMLP-activated MDSC from transplanted patients inhibited T cell proliferation (41). MDSC were shown to expand early after transplantation, independently of using basiliximab or thymoglobulin during induction (42) (and our unpublished observations).

Consistent with data from Vanhove's laboratory describing the presence of MDSC and of Treg in kidney transplanted rats, data from Murphy and colleagues reported presence of MDSC in human kidney transplant recipients. The study evaluated the capacity of blood derived CD11b^+^CD33^+^HLA-DR^−^ MDSC to suppress CD4^+^T cells proliferation *in vitro*. In addition, blood derived MDSC were able to expand Treg *in vitro* and correlated with increased Treg numbers *in vivo* (43). This was the first study where MDSC were associated with Treg in human transplant recipients. Consistent with these results, *ex vivo* experiments performed by Hoechst and colleagues demonstrated that blood derived CD14^+^HLA-DR^−−/^ myeloid cells isolated from hepatocellular carcinoma (HCC) patients induce CD4^+^CD25^+^Foxp3^+^ Treg when co-cultured with autologous T cells (44). A recent report from this group demonstrated that, while CD14^+^HLA-DR^−^ MDSC induce Foxp3^+^ Treg, CD14^+^HLA-DR^+^ myeloid cells from the same patient promote the generation of pathogenic Th17 cells when co-cultured with naive CD4^+^ T cells. Importantly CD14^+^HLA-DR^−^ MDSC modulate the trans differentiation of Foxp3^+^ Treg from monocyte-induced Th17 cells in a TGF-β and retinoic acid (RA) dependent mechanism (45).

More recently, the expression of myeloid-related S100A8 and S100A9 proteins was analyzed in two independent cohorts of patients with acute rejection. These proteins showed *in vitro* suppressive properties including inhibition of DC maturation and enhancement of ROS production. High S100A8 and S100A9 mRNA levels in biopsies predicted improved graft outcome and both proteins expression correlated with MDSC markers into PBMC and renal biopsies. Intragraft, high amounts of S100A9 correlated with lower expression of T cell immunity (CD3ε and CD4) and increased FoxP3, IL-10, and TGF-β regulatory markers (46). In a retrospective cohort of 50 renal recipients with biopsy-proven acute T-cell mediated rejection, patients with high MDSC in circulation (CD11b^+^CD33^+^HLA-DR^−^ cells) (MDSC > 10% into PBMC) showed increased estimated glomerular filtration rate and lower serum creatinine at the time of biopsy. Compared to low MDSC recipients, high MDSC patients showed less development of IFTA and significantly superior 1- and 5-year graft survival (47). However, prospective and randomized studies in large cohorts are still lacking to better understand the role of MDSC in clinical kidney transplant and their potential value as biomarker or therapy target.

### Corneal Transplantation

Corneal allograft models in mice have been used to test the use or manipulation of MDSC as immunomodulatory strategies in transplantation. In two groups of mice receiving either cornea or cornea and skin allografts, longer grafts survival was observed in animals with prior transference of bone marrow MDSC induced in cecal ligated and punctured mice (48). In a different experimental model, B6 mice corneas were transplanted into BALB/c recipients who received intraperitoneal dexamethasone (dex) at decreasing doses from day 0 to 21 after surgery. Administration of dex significantly prolonged the allograft survival and correlated with decreased infiltration of CD3+ cells and low levels of IFN-γ and IL-1β in the grafts, together with low IFN-γ CD4+ cells in draining lymph nodes, blood, spleen and bone marrow. Concomitantly, an expansion of MHC class II^−^CD11b^+^Ly6C^+^ monocytes (m-MDSC following Bronte and coworkers, ref 6) and increased iNOS were observed in the same compartments of dex-treated mice. FACS-sorted CD11b^+^Ly6C^+^ cells from bone marrow of dex-treated mice inhibited *in vitro* CD4+ T cells proliferation and prolonged the survival of corneal allografts when transferred into dex-untreated recipients. Depletion of MHC class II^−^CD11b^+^Ly6C^+^ monocytes abrogated the protective effect of dex on corneal allografts suggesting that these cells were required for mediating the induction of tolerance by glucocorticoids (49). Similar results were obtained in corneal allograft recipient mice treated with a rapamycin nano-micelle (RNM) ophthalmic solution. Under this therapy, delay of rejection and expansion of Gr1^int^ CD11b^+^ MDSCs in allografts, cervical lymph nodes, blood and spleen were observed. The capacity of MDSCs from the RNM solution-treated mice to suppress proliferation of CD4+ T cells depended on iNOS and arginase-1, and the administration of anti-Gr-1 antibody or the pharmacological inhibition of iNOS abrogated the beneficial effects of rapamycin (50).

### Pancreatic Islets Transplantation

In the Bronte's pioneer work murine MDSCs were obtained by treating bone marrow *in vitro* with GM-CSF + IL-6. When adoptively transferred into islet-allografted syngeneic mice, the GM-CSF + IL-6 induced MDSCs increased the survival of functional islets. Integrity of the C/EBPβ transcription factors was needed to develop the tolerogenic MDSC (51).

In different experimental settings, prolonged islets allograft survival has been achieved by MDSC generated by hepatic stellate cells (HSC). CCR2 expression was needed to allow migration of HSC-induced MDSC into allografts (52), and their suppressive capacity relied on induction of apoptosis in T effector cells and B7-H1-mediated expansion of Tregs (53, 54). The induction of MDSC by HSC depended from soluble factors. Interestingly, SDS-PAGE and LC-MS analysis of the most bioactive fraction in hepatic stellate cells culture supernatant identified complement component 3 (C3) as a key mediator, since depletion of HSC-derived C3 markedly reduced the ability to induce MDSC (53). Moreover, co-transplantation of BALB/c mice islets with wild type or C3^−/−^ HSC into diabetic B6 mice showed that 60% of mice receiving wild type HSC remained normoglycemic by post-transplant day 60 while all recipients of C3-deficient HSC lost islets under day 21. Draining lymph nodes and grafts from wild type HSC recipients showed increased frequency of Tregs and CD11c^−^ myeloid cells of immature phenotype, which were able to suppress proliferation of CD4^+^ and CD8^+^ cells and production of IFNγ (55). However, results in the opposite direction were obtained in a model of streptozotocin-induced diabetes in mice, in which expansion of MDSC and protection of pancreas against the autoimmune destruction were noticed in the absence of C3, and depletion of MDSC by anti-Gr1 led to diabetes in C3^−/−^ streptozotocin-treated mice (56). Concluding results to fully understand if and how C3 is required to promote generation of MDSC are still missing.

### Skin Transplantation

Using an experimental skin transplant model where bm12 MHC-II minor mismatched graft were transplanted into C57BL/6 recipients, the Horuzsko laboratory demonstrated HLD-G expressing MDSC interacts with immune inhibitory receptors, such as immunoglobulin-like transcript 2 (ILT2), which induces the expansion of MDSC *in vivo* (57). The authors further demonstrated that MDSC from ILT2 transgenic mice exhibit an augmented suppressive function and were able to prolong skin graft survival following adoptive transfer into C57BL/6 recipients.

De Wilde and colleagues demonstrated that repetitive administration of lipopolysaccharide (LPS) *in vivo* induces the development CD11b^+^Gr-1^+^ MDSC (58). In a male-to-female mismatched skin transplantation model, the authors reported that *in vivo* transfer of MDSC treated with LPS significantly prolonged skin allograft survival. This was due to the expression of heme oxygenase-1 (HO-1), which impaired T cell activation. The authors also demonstrated that LPS induced MDSC suppresses Th1 and Th2 cytokine production, while produce large amounts of IL-10 as suppressive mechanism. Further, HO-1 inhibition by a specific inhibitor completely abolished T-cell suppression and IL-10 production. The importance of HO-1 during prolonged allograft survival was first described by Yamashita et al. (59). The authors demonstrated that cobalt protoporphyrin IX (CoPPIX) treatment leads to a significant up-regulation HO-1 that was necessary for indefinite survival of fully mismatched heart allografts.

In a recent study, Zhao et al. established that the combination of M-CSF and TNFα efficiently induces functional MDSC *in vitro* (60). The resulting M-MDSC were characterized by the expression of F4/80, CD80, and PD-L1. Mechanistically, M-CSF + TNFα induced M-MDSC upregulated the expression of iNOS, which was necessary for suppression of T cell proliferation. Upon adoptive transfer, M-CSF+TNFα induced M-MDSC promoted immune tolerance in male-to-female skin transplanted mice. Consistent with Vanhove's observation, blockade of iNOS activity failed to induce the graft acceptance, demonstrating that immunosuppressive ability of M-CSF+TNFα-induced M-MDSC is dependent on iNOS. The critical role of iNOS activity in the suppressive function of MDSC was also described by Wu et al. (61). The authors identified Smad3 as an intrinsic negative regulator of MDCS development and recognized that the immunosuppressive function of MDCS depends on NO production. Using *Smad3* deficient mouse recipients in a fully mismatched skin transplantation model, the authors observed an increase in both granulocytic and monocytic cells associated with less production of anti-donor IgG Abs and decreased IFN-γ production. Interestingly, L-NMMA significantly reduced NO production and efficiently blocked the immunosuppressive effects of Smad3-deficient G-MDSC on T cell proliferation.

In agreement with these results, Liao and colleagues demonstrated that MDSC development is induced by dexamethasone through the glucocorticoid receptor (GR) pathway (62, 63). Dexamethasone treatment significantly prolonged allograft survival in a fully allogeneic skin transplant model in mice through upregulation of iNOS and NO production in MDSC. These results validate the administration of glucocorticoids as a therapeutic approach that prolongs graft survival through the development of MDSC.

Further studies have also illustrated the immune-modulatory activity of IL-33 during the induction of iNOS expressing MDSC (64). Using both syngeneic and allogeneic skin transplants models, Pino-Lagos and colleagues demonstrated that IL-33 treatment up-regulated the number of Foxp3^+^ Treg and promoted the conversion of Foxp3^−^ T cells into Foxp3^+^ Treg in the periphery.

### Heart Transplantation

Using a mouse heart transplantation model Rodriguez-Garcia and colleagues demonstrated the requirement of MDSC for the induction of transplantation tolerance (65). The authors treated recipient mice with anti-CD40L mAb costimulatory blockade and identified the critical role for tolerogenic CD11b^+^Ly6C^low^ expressing MDSC. Using depletional mAbs, clodronate-loaded liposomes, and transgenic mice specific for depletion of CD11b^+^ expressing cells the authors reported that monocytic precursors migrate from the bone marrow to the transplanted organ early after transplantation and prevent the initiation of adaptive immune responses that lead to allograft rejection.

Similar results were previously obtained from Terry Strom laboratory (66), which demonstrated that CD11b^+^Gr1^low^ MDSC exhibit high suppressive capacities and prevent grafts from prolonged cold ischemia-mediated injury. The authors induced the development of Ly6C^low^ MDSC by treating fully allogeneic recipient mice with rapamycin (3 mg/kg) and costimulatory blockade with anti-CD40L mAb. This combination therapy induced tolerance in C57BL/6 mice pre-sensitized by Balb/c skin grafts at day-7 that received BALB/c heart grafts in contrast to mice treated with either rapamycin or anti-CD40L mAb alone.

Consistent with these results, a recent report from Braza and colleagues described a novel nanoimmunotherapy based on high-density lipoprotein (HDL) that targets myeloid cell precursors *in vivo* (32). Using a fully allogeneic mouse heart transplant model, the authors demonstrated that a rapamycin and CD40 costimulatory blockade combined nanotherapy (mTORi/Traf6i-HDL) favored the accumulation of CD11b^+^Ly6C^low^ myeloid cells, which prevented CD8^+^ T cell proliferation and promoted Treg expansion. Remarkably, a short-term treatment with nanobiologics during the first week after transplantation resulted in indefinite allograft survival of most transplant recipients with no signs of chronic rejection.

Considering mTOR inhibitors as a current immunosuppressive therapy for organ transplantation, Nakamura and colleagues demonstrated that treatment with rapamycin results in a significant prolongation of graft survival mediated by iNOS expressing MDSC in a murine cardiac transplantation model (67). The authors also confirmed that CD11b^+^ myeloid cells expressing lower levels of Gr-1 efficiently suppressed CD4^+^ T cell proliferation *in vitro*. Interestingly, adoptive transfer of rapamycin-induced MDSC and to a lesser extent G-MDSC, through the coronary arteries before organ reperfusion of transplant recipient mice significantly prolonged allograft survival. Graft survival prolongation of MDSC was associated with an increase of splenic Foxp3^+^ Treg. Mechanistically, the Nakamura group further reported that rapamycin treatment induces the expression of PD-L1 on MDSC that accumulate in the cardiac allograft following adoptive transfer (68).

A critical aspect in organ transplantation is the induction of donor-specific unresponsiveness. For this purpose, many authors use donor specific splenocytes in combination with tolerogenic therapy, such as co-stimulatory blockade (69, 70). In this respect, Luo and colleagues performed infusions of donor splenocytes treated with 1-ethyl-3-(3′-dimethylaminopropyl)-carbodiimide (ECDI-SPs) before and after transplantation (71, 72). The authors observed prolonged allograft survival associated with intragraft accumulation of CD11b^+^ MDSC that express high levels of indoleamine 2,3 dioxygenase (IDO). Furthermore, combination therapy of donor ECDI-SPs with systemic rapamycin induced indefinite cardiac allograft survival in 100% of the recipients for over 150 days.

### Liver, Bowel, and Lung Transplantation

Whereas, liver transplants can be spontaneously accepted without the requirement of immunosuppression in different species (73, 74), the immune response acutely rejects hepatocyte transplants (75). This suggests that liver stromal cells protect parenchymal cells from rejection. Hepatic stellate cells have potent immune regulatory activity and they have been shown to promote MDSCs generation *in vivo* and *in vitro* (53, 76).

Kim et al. showed that in Rhesus macaques MDSC accumulate in high numbers in the liver when compared to blood, spleen and lymph nodes (77). In a model of allogeneic orthotopic liver transplantation in rats, the authors observed that the promotion of tolerance by treatment with rapamycin was associated with an increase of regulatory T cell phenotypes and accumulation of MDSC in spleen (78).

In a prospective cohort of 36 intestinal transplant recipients, the authors identified MDSC (lineage^−^HLADR^−/low^CD33^+^CD11b^+^-expressing cells) and all three M- (CD14^+^CD15^−^), PMN- (CD14^−^CD15^+^) and e-MDSC populations into PBMC. All three MDSCs subsets increased post-transplant although PMN-MDSC and e-MDSC did so immediately, while M-MDSC increased after 2 months post-transplant. All three MDSC types were able to suppress CD4+ and CD8+ T cells proliferation as well as IFNγ production. High plasma levels of IL-6 but not TNFα or GM-CSF, the use of exogenous steroids and low tacrolimus trough levels correlated with MDSCs numbers in PBMC. In agreement, IL-6 and methylprednisolone enhanced MDSC cells after culturing bone marrow cells from healthy controls in basic medium with GM-CSF and G-CSF. Intragraft MDSCs were low before transplantation but increased during a year after transplantation. The analysis of chemokines expression in intestinal grafts biopsies and of chemokines receptors expression in MDSC supported a role for CCL11 and CCL15 in recruiting CCR1- and CCR3- expressing M- MDSCs and e-MDSCs, and a role for CXCL6 in recruiting CXCR2- expressing PMN- MDSCs and e- MDSCs into the mucosa of intestinal allografts. Peripheral blood MDSCs were significantly lower in patients with acute rejection. The addition of MDSC into co-cultures of donor-reactive T cells with donor-derived intestinal epithelial organoids enhanced the organoids viability, suggesting that the accumulation of MDSC suppressed T cells alloresponse against the donor intestinal epithelium (79).

In the only publication regarding lung transplant at present, the authors observed that the phyla *Firmicutes* dominated the microbiome signature in the distal airways of subjects without bronchiolitis obliterans syndrome (BOS), while this shifted to a Proteobacteria-dominant signature in the BOS cohort. Suppressive MDSC predominated in the proximal airways and pro-inflammatory myeloid cells were more abundant in distal airways. These results suggested a functional link between the local microbiome and MDSC phenotype, which may play a role in the pathogenesis of BOS (80).

## Conclusion and Perspectives

Regulation of different MDSC subsets with distinct immune function may be used for future therapeutic approach that promotes tolerance in organ transplantation. In the setting of murine GVHD, the inflammasome activation in the transferred MDSC induced the loss of their suppressive capacity, thus, understanding the micro environmental signals that affect the stability of the MDSC suppressive capacity will be also critical for an optimal use in therapy (81). In addition, MDSC may be used as biomarkers that provide critical information regarding the functional immune status of organ transplant recipients. Non-invasive immune approaches that determine and characterize MDSC subsets in humans are urgently needed to move the field forward. Unfortunately, identification of specific mechanisms by which MDSC exhibit suppressive functions and contribute to the development of tolerance remains a difficult task. One of the main difficulties resides in a consensual classification and identification MDSC subset during pathological conditions. For that purpose, the COST action Mye-EUNITER was established in 2014 (http://www.mye-euniter.eu/) to create a general consensus to standardize the function and phenotype of MDSC across different species (82). Additional immune regulatory molecules, such as B7-H3 (83), may be validated in future experiments to further identify and characterize MDSC in transplant recipients. In this respect, the use of next generation genomic sequencing may help to identify the transcriptomic profile of different MDSC subsets and differentiate suppressor cells from normal myeloid cells (84).

One critical consideration to fully determine whether MDSC are indeed functionally inhibitory myeloid cell is the choice of the immune functional assay (85). While the clinical application of MDSC represents a promising therapeutic approach for the induction of organ transplant acceptance either as a cell therapy or by regulating there *in vivo* development from myeloid precursors, it requires consensus on markers that identify MDSC subsets, which limit our ability to specifically target MDSC *in vivo*.

## Author Contributions

All authors listed have made a substantial, direct and intellectual contribution to the work, and approved it for publication.

### Conflict of Interest Statement

The authors declare that the research was conducted in the absence of any commercial or financial relationships that could be construed as a potential conflict of interest.
